# Preliminary Results of MyCog, a Brief Assessment for the Detection of Cognitive Impairment in Primary Care

**DOI:** 10.1093/geroni/igaa057.833

**Published:** 2020-12-16

**Authors:** Laura Curtis, Lauren Opsasnick, Julia Yoshino Benavente, Cindy Nowinski, Rachel O’Conor, Jordan Stoeger, Michael Wolf, Richard Gershon

**Affiliations:** Northwestern University, Chicago, Illinois, United States

## Abstract

Early detection of Cognitive impairment (CI) is imperative to identify potentially treatable underlying conditions or provide supportive services when due to progressive conditions such as Alzheimer’s Disease. While primary care settings are ideal for identifying CI, it frequently goes undetected. We developed ‘MyCog’, a brief technology-enabled, 2-step assessment to detect CI and dementia in primary care settings. We piloted MyCog in 80 participants 65 and older recruited from an ongoing cognitive aging study. Cases were identified either by a documented diagnosis of dementia or mild cognitive impairment (MCI) or based on a comprehensive cognitive battery. Administered via an iPad, Step 1 consists of a single self-report item indicating concern about memory or other thinking problems and Step 2 includes two cognitive assessments from the NIH Toolbox: Picture Sequence Memory (PSM) and Dimensional Change Card Sorting (DCCS). 39%(31/80) participants were considered cognitively impaired. Those who expressed concern in Step 1 (n=52, 66%) resulted in a 37% false positive and 3% false negative rate. With the addition of the PSM and DCCS assessments in Step 2, the paradigm demonstrated 91% sensitivity, 75% specificity and an area under the ROC curve (AUC)=0.82. Steps 1 and 2 had an average administration time of <7 minutes. We continue to optimize MyCog by 1) examining additional items for Step 1 to reduce the false positive rate and 2) creating a self-administered version to optimize use in clinical settings. With further validation, MyCog offers a practical, scalable paradigm for the routine detection of cognitive impairment and dementia.

